# Chemical Chaperones Modulate the Formation of Metabolite Assemblies

**DOI:** 10.3390/ijms22179172

**Published:** 2021-08-25

**Authors:** Hanaa Adsi, Shon A. Levkovich, Elvira Haimov, Topaz Kreiser, Massimiliano Meli, Hamutal Engel, Luba Simhaev, Shai Karidi-Heller, Giorgio Colombo, Ehud Gazit, Dana Laor Bar-Yosef

**Affiliations:** 1 Shmunis School of Biomedicine and Cancer Research, George S. Wise Faculty of Life Sciences, Tel Aviv University, Tel Aviv 6997801, Israel; hanaaadsi@mail.tau.ac.il (H.A.); shonl@tauex.tau.ac.il (S.A.L.); topazk136@gmail.com (T.K.); 2 BLAVATNIK CENTER for Drug Discovery, Tel Aviv University, Tel Aviv 6997801, Israel; haimove@tauex.tau.ac.il (E.H.); taliengel@tauex.tau.ac.il (H.E.); luba.simhaev@gmail.com (L.S.); 3 SCITEC-CNR, via Mario Bianco 9, 20131 Milano, Italy; massimiliano.meli@gmail.com (M.M.); g.colombo@unipv.it (G.C.); 4 The Future Scientists Center–Alpha Program at Tel Aviv Youth University, Tel Aviv 6997801, Israel; shaisacc@gmail.com; 5 Department of Chemistry, University of Pavia, via Taramelli 12, 27100 Pavia, Italy; 6 Department of Materials Science and Engineering, Iby and Aladar Fleischman Faculty of Engineering, Tel Aviv University, Tel Aviv 6997801, Israel

**Keywords:** chemical chaperones, osmolytes, hydrophobic compounds, metabolite assemblies, amyloid formation, adenine, inborn errors of metabolism

## Abstract

The formation of amyloid-like structures by metabolites is associated with several inborn errors of metabolism (IEMs). These structures display most of the biological, chemical and physical properties of protein amyloids. However, the molecular interactions underlying the assembly remain elusive, and so far, no modulating therapeutic agents are available for clinical use. Chemical chaperones are known to inhibit protein and peptide amyloid formation and stabilize misfolded enzymes. Here, we provide an in-depth characterization of the inhibitory effect of osmolytes and hydrophobic chemical chaperones on metabolite assemblies, thus extending their functional repertoire. We applied a combined in vivo-in vitro-in silico approach and show their ability to inhibit metabolite amyloid-induced toxicity and reduce cellular amyloid content in yeast. We further used various biophysical techniques demonstrating direct inhibition of adenine self-assembly and alteration of fibril morphology by chemical chaperones. Using a scaffold-based approach, we analyzed the physiochemical properties of various dimethyl sulfoxide derivatives and their role in inhibiting metabolite self-assembly. Lastly, we employed whole-atom molecular dynamics simulations to elucidate the role of hydrogen bonds in osmolyte inhibition. Our results imply a dual mode of action of chemical chaperones as IEMs therapeutics, that could be implemented in the rational design of novel lead-like molecules.

## 1. Introduction

Proteins are highly functionally and structurally diverse biomolecules. To function properly, proteins usually obtain a specific conformational state that is considered ‘native’. However, the folded state of proteins is often only marginally stable [1,2,3]. Certain genetic, physiological, and environmental conditions can induce protein misfolding, resulting in the formation of small aggregation-prone species that tend to self-assemble into supramolecular assemblies [4]. In some cases, these assemblies obtain a fibrillar morphology and a set of canonical biophysical and biochemical traits, which are the hallmarks of amyloids [4,5]. Amyloid formation is associated with a wide range of disorders, such as type 2 diabetes, Alzheimer’s disease, and Parkinson’s disease, and is thus considered a central target for therapeutic intervention [4].

Chaperones play a pivotal role as the safeguards of the protein quality control system and take part in enhancing the proper folding of proteins, thus counteracting the potentially deleterious effect of protein misfolding [6]. They are classified into three main groups: molecular, pharmacological, and chemical chaperones [7]. Molecular chaperones (such as heat shock proteins) facilitate the proper folding of other proteins and are central players in the proteostasis network [6,7,8,9]. Pharmacological chaperones are small molecules that specifically bind misfolded proteins and induce refolding or structure stabilization [10]. Unlike pharmacological chaperones, chemical chaperones are small molecules that do not bind a single misfolded protein-ligand but rather function in a non-specific manner [10]. Chemical chaperones are divided into two structurally and functionally distinct groups: osmolytes and hydrophobic compounds [11]. Osmolytes are low molecular weight compounds such as amino acids, polyols, and methylamines (e.g., glycine, glycerol, dimethyl sulfoxide (DMSO), and trimethylamine N-oxide (TMAO)). Osmolytes increase the stability of the folded state by sequestering water molecules, thereby generating a preferential depletion of the osmolyte adjacent to the protein surface, leading to protein stabilization and conformational changes [12]. Hydrophobic compounds include several bile acids (e.g., deoxycholic acid (DCA) and ursodeoxycholic acid (UDCA)). Their mode of action is postulated to include direct interactions with the hydrophobic regions of misfolded proteins, thus favoring the folded form. However, the exact mechanism of action of this group is still not fully understood [11].

Chemical chaperones were shown to reduce endoplasmic reticulum stress, correct the misfolded form of the p53 tumor suppressor protein and restore glucose homeostasis [13,14,15,16,17]. The effect of different chemical chaperones in ameliorating protein misfolding in neurodegenerative and prion diseases has been well documented both in vitro and in vivo [18,19,20,21,22,23,24,25]. Chemical chaperones thus could potentially serve as scaffolds for the development of novel therapeutics for protein misfolding diseases.

**Figure 1:**
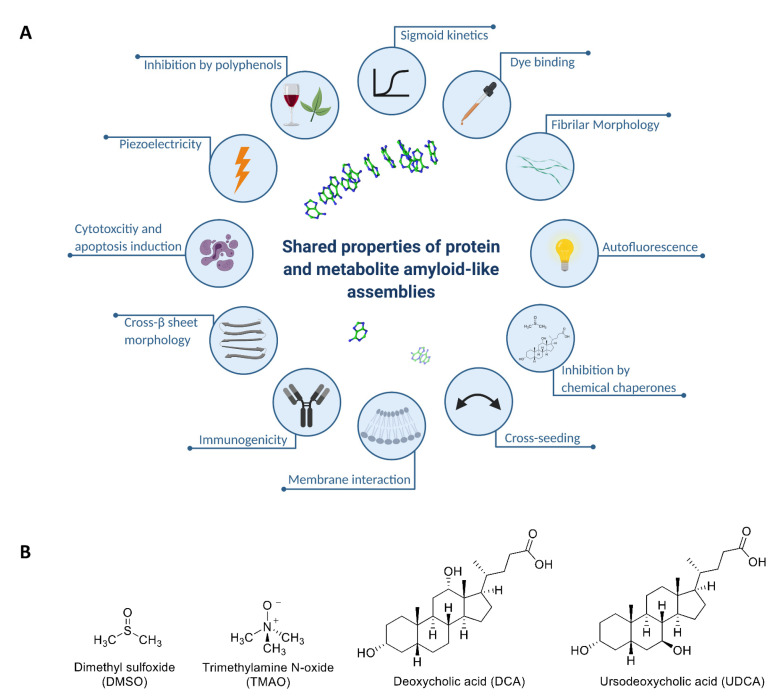
(**A**) Illustration of the shared properties of metabolite amyloid-like structures and protein or peptide amyloids: nanoscale fibrillar morphology [33,34,35,36], β-sheet secondary structure [37], amyloid-specific fluorescent dye binding [27,33,34], cytotoxicity and apoptosis induction [27,33,34,35,36,38,39], membrane interaction [40,41,42,43,44], inhibition by polyphenols [27,45], cross-seeding with protein amyloids [36,46], immunogenicity [27,33,36,47], intrinsic fluorescence [48,49], sigmoid growth curve [27], piezoelectricity [50], and inhibition by chemical chaperones (this study). Created with BioRender.com. (**B**) Molecular structure of DMSO, TMAO, DCA, and UDCA.

A reductionist approach has allowed to re-define the minimal building block required for amyloid formation, demonstrating that this phenomenon is not restricted to proteins and peptides. Specifically, we have established that metabolites such as amino acids, nucleobases, and other small molecules can self-assemble into well-ordered amyloid-like fibrillar structures that share many common biophysical and biochemical properties with classical protein amyloids (Figure 1A). The extension of the canonical amyloid hypothesis has provided a novel paradigm for the etiology of human inborn errors of metabolism (IEMs), wherein a congenital enzymatic deficiency results in the accumulation of one or more metabolites [26]. We have recently established a salvage yeast model for adenine accumulation and self-assembly by recapitulating the enzymatic deficiency of patients suffering from metabolic disorders associated with the accumulation of adenine and its derivatives [27]. This model demonstrates robust sensitivity to adenine that is attributed to the formation of toxic adenine amyloid-like supramolecular structures, as indicated by staining with an amyloid-specific dye and specific antibodies raised against the fibrils. These effects were decreased upon treatment with generic fibrillation-modifying polyphenolic compounds such as tannic acid, thus supporting the potential of the model as a drug discovery platform [27,28]. 

While the effect of molecular and chemical chaperones on misfolded proteins and the formation of proteinaceous amyloid assemblies is well characterized [6,29,30], it is unknown whether similar chaperoning mechanisms affect metabolite self-assembly. In this study, we applied a multimodal in vivo-in vitro-in silico approach to characterize the effect of chemical chaperones on metabolite self-assembly and amyloid formation. For this purpose, we examined the effect of four naturally occurring representative chemical chaperones: the DMSO osmolyte (found in various fruits, vegetables, grains, and beverages), TMAO osmolyte (one of the gut-derived metabolites found in diverse marine organisms), and the hydrophobic bile acids DCA and UDCA (Figure 1B) [11,31,32]. We report their ability to reduce metabolite amyloid content and associated toxicity in yeast, as well as to inhibit the formation of adenine amyloid-like structures and alter their morphology in vitro. We further aimed to evaluate the potential of chemical chaperones as a basis for the rational design of potent lead-like molecules. We have thus applied structure–activity and structure-property relationship (SAR-SPR) analysis to examine the effect of various DMSO derivatives on adenine fibrillization, revealing the importance of hydrogen bonds for effective inhibition. This notion was supported by molecular dynamics (MD) simulations which illustrated the formation of hydrogen bonds between adenine and the lead-like sulfoxide derivative we have identified. Lastly, we report a common ‘hijacking’ mechanism, by which both hydrophobic chaperones and osmolytes interfere with the stacking of the adenine molecules, thus inhibiting metabolite self-assembly. 

## 2. Results

### 2.1. Chemical Chaperones Suppress Adenine Toxicity and Inhibit Intracellular Amyloid Formation in a Mutant Yeast Salvage Model

**Figure 2:**
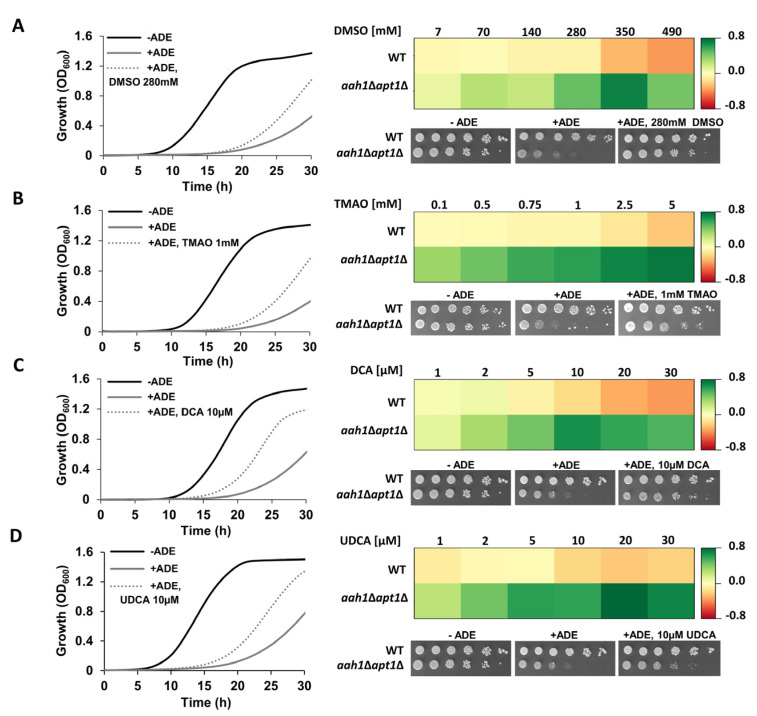
Inhibition of adenine toxicity by chemical chaperones in a yeast model. **Left panel:** Growth curves of *aah1*Δ*apt1*Δ cells under the indicated conditions, at selected chaperones concentrations (see Supplementary Figure S1 for full data). Cells were grown at 30 °C and the absorbance at OD_600_ was measured over time (see Material and Methods). The results are representative of three independent biological repeats. **Right panel:** Dose-response heat maps representing the difference in optical density between a chaperone-supplemented culture and the control condition (+ADE) when the control cells reached OD_600_ = 0.6. The dose-response heat maps were generated based on the data presented in Supplementary Figure S1. Green, rescue. Red, toxicity. WT and *aah1*Δ*apt1*Δ yeast were serially diluted and spotted on synthetic defined (SD) media lacking adenine (−ADE), SD media with adenine supplemented at 2 mg/L as a control for DMSO and TMAO, or with 2 mg/L adenine and 0.1% DMSO as a control for DCA and UDCA (+ADE), and SD with 2 mg/L adenine and the indicated concentration of the chemical chaperone. (**A**) DMSO; (**B**) TMAO; (**C**) DCA; (**D**) UDCA.

We first utilized our established salvage yeast model of adenine self-assembly (*aah1*Δ*apt1*Δ) to examine the effect of DMSO, TMAO, DCA, and UDCA on phenotypes associated with adenine toxicity [27]. In line with their reported ability to inhibit protein and peptide amyloid formation [11], we observed a significant dose-dependent rescue of the adenine toxicity in yeast (Figure 2 and Supplementary Figure S1). 

**Figure 3:**
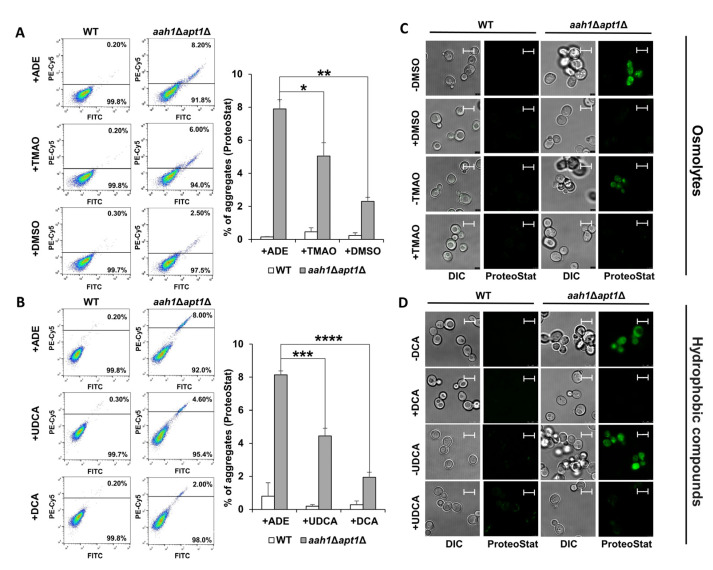
Chemical chaperones inhibit adenine aggregation in the salvage yeast model. (**A**,**B**) Flow cytometry analysis of wild type (WT) and *aah1*Δ*apt1*Δ yeast cells under the indicated conditions following ProteoStat staining. Left: Flow cytometry data. Right: Quantification of ProteoStat positive cells under the indicated conditions. * *p* < 0.05; ** *p* < 0.005; *** *p* < 0.001; **** *p* < 0.0001 (Student’s *t*-test). Values are the average ± s.d. of three independent biological repeats. (**A**) 2 mg/L adenine (+ADE), 2 mg/L adenine and 1 mM TMAO (+TMAO) or 140 mM DMSO (+DMSO). (**B**) 2 mg/L adenine containing 0.05% DMSO (+ADE), 2 mg/L adenine containing 0.05% DMSO, 10 µM UDCA (+UDCA) or 10 µM DCA (+DCA). (**C**,**D**) Representative confocal microscopy images of WT and *aah1*Δ*apt1*Δ cells under the same conditions as in (**A**,**B**). Scale bar is 5 μm.

In order to examine whether the chaperone-induced growth improvement correlates with inhibition of amyloid aggregation, we stained the cells with the ProteoStat amyloid-specific dye, previously demonstrated to stain both protein and metabolite amyloids in vivo [27,51]. Flow cytometry and confocal microscopy were applied to visualize and analyze the intracellular amyloid content. As previously reported, adenine supplementation to the salvage mutant dramatically increased the intracellular amyloid content (Figure 3A,B), and resulted in a punctate staining pattern (Figure 3C,D) [27]. In accordance with the growth assays (Figure 2), the supplementation of chemical chaperones resulted in a significant reduction in the intracellular aggregate content in the mutant strain (Figure 3A,B). Furthermore, treatment with the chaperones significantly reduced the intracellular staining intensity (Figure 3C,D). 

### 2.2. Chemical Chaperones Alter the Biophysical Properties of Adenine Amyloid-like Assemblies 

**Figure 4:**
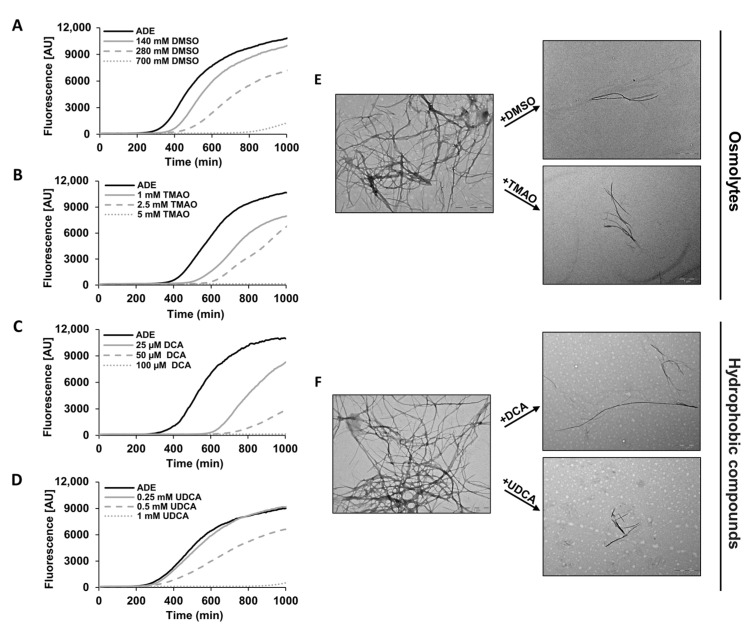
Inhibition of adenine fibril formation by different chemical chaperones in vitro. (**A**–**D**) Kinetic analysis of adenine aggregation as measured in a ThT binding assay. Adenine was dissolved at 6 mg/mL in PBS and heated to 90 °C to obtain a monomeric solution, followed by the addition of the chemical chaperones at the indicated concentrations (see Materials and Methods). ThT was added to a final concentration of 40 µM, and fluorescence was measured over time with excitation and emission wavelengths of 450 nm and 480 nm, respectively. The results are representative of three independent repeats. (**E**,**F**) Representative TEM micrographs of 1 mg/mL adenine in the presence or absence of the indicated chemical chaperone at 1.4 mM DMSO, 0.1 mM TMAO, 50 µM DCA, and 50 µM UDCA. Scale bar is 1 µm. The images are representatives of three double-blinded repeats.

We next aimed to elucidate the mechanism of action underlying the effect of chemical chaperones on the cellular phenotypes investigated (Figure 2 and Figure 3). Therefore, we examined whether chemical chaperones directly impede adenine self-assembly in vitro by monitoring the binding kinetics of the fluorescent amyloid-specific dye Thioflavin-T (ThT). In line with the yeast assays data, all the tested chemical chaperones significantly reduced the adenine self-assembly rates in a dose-dependent manner, as demonstrated by inhibition of ThT binding (Figure 4A–D). Similar results were obtained using ProteoStat, providing the first demonstration of its utilization for staining metabolite amyloids in vitro (Supplementary Figure S2). The reduction in the ProteoStat fluorescence in a dose-dependent manner, reinforces the notion that chemical chaperones possess the ability to inhibit adenine self-assembly (Supplementary Figure S2).

We then aimed to characterize the effect of chemical chaperones on the morphology of the adenine amyloid-like fibrils using transmission electron microscopy (TEM). While typical fibrillar structures were observed in the control samples, the supplementation of chemical chaperones significantly reduced the prevalence of the fibrils and rendered them shorter and less densely packed (Figure 4E,F). These observations align with the results obtained from the cytotoxicity assays, further supporting the potency of chemical chaperones as inhibitors of adenine self-assembly. 

### 2.3. Structure–Activity and Structure–Properties Relationships of DMSO Derivatives 

**Figure 5:**
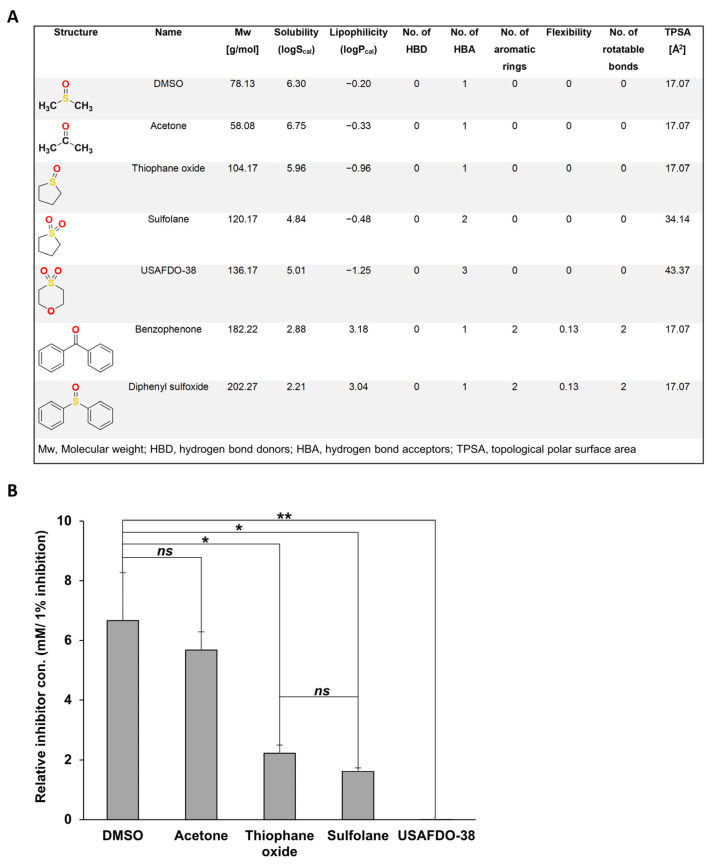
The effect of different DMSO derivatives on adenine self-assembly inhibition. (**A**) Physicochemical properties of different DMSO derivatives calculated by StarDrop (Optibrium). Oxygen atoms are shown in red; sulfur atoms are shown in yellow. (**B**) Kinetic analysis of adenine aggregation as measured in a ThT binding assay. Adenine was dissolved at 6 mg/mL in PBS and heated to 90 °C to obtain a monomeric solution, followed by the addition of DMSO or its derivatives at the indicated concentrations (see Supplementary Figure S3). The controls for each compound were prepared as described in the Materials and Methods. ThT was added to a final concentration of 40 µM, and fluorescence was measured over time with excitation and emission wavelengths of 450 nm and 480 nm, respectively. The relative concentration was calculated at an endpoint of 1000 min, and the concentration of the inhibitor was divided by the percentage of inhibition. The relative concentration represents the minimal concentration required to inhibit 1% of adenine fibrilization (relative con. (mM/1% inhibition)) (see material and method section and supplementary Figure S3). ns, not significant; * *p* < 0.02; ** *p* < 0.01 (Student’s *t*-test). Values are the average ± s.d. of three independent biological repeats.

Next, we sought to examine whether chemical chaperones can serve as structural scaffolds for the rational design of more potent inhibitors. We chose DMSO as a model molecule, owing to its low molecular weight (78.13 (g/mol)) and structural simplicity. We rationally selected several DMSO derivatives based on their molecular structure and physicochemical characteristics typically associated with ‘drug-like’ properties: molecular weight, lipophilicity (logP_cal_), aqueous solubility (logS_cal_), number of hydrogen bond donors (HBD), number of hydrogen bond acceptors (HBA), number of aromatic rings, flexibility, and number of rotatable bonds and topological polar surface area (TPSA) (Figure 5A) [52]. 

We then examined the inhibition potency of the candidate molecules in vitro, aiming to study the implications of the SAR-SPR analysis [53,54,55]. The inhibition potency of the molecules was measured by calculating the relative concentration needed to inhibit 1% adenine aggregation (Figure 5B). Acetone, which has similar physicochemical properties to DMSO and differs only in the quaternary atom (sulfur replaced to carbon), showed no significant difference in the relative concentration when compared to DMSO (Figure 5B and Supplementary Figure S3A,B). On the other hand, tetrahydrothiophene 1-oxide (thiophane oxide), the cyclic derivative of DMSO, exhibited more than three times lower relative concentration for inhibition. Thiophane oxide and tetrahydrothiophene 1,1-dioxide (sulfolane) differ in one conjugated HBA. However, the relative concentration of sulfolane is lower than thiophane oxide, thus displaying a slightly higher inhibition potency (Figure 5B and Supplementary Figure S3C,D). Remarkably, the presence of a non-conjugated HBA in 1,4-thioxane-1,1-dioxide (USAFDO-38) significantly increased the efficacy compared to DMSO as displayed by the significant decrease in the relative concentration of the compound (Figure 5B and Supplementary Figure S3C–E). 

The SAR-SPR analysis of these derivatives marks HBA as the physicochemical property most significantly contributing to the potency of the compound at inhibiting adenine self-assembly. Notably, the number of hydrogen bond donors and acceptors corresponds to higher TPSA, which is known to directly correlate with the specificity and safety of a designed drug [56,57]. Hence, USAFDO-38 not only shows the lowest relative concentration but also has a promising molecular framework attributed to its physicochemical properties, and thus could serve as a promising scaffold for future studies (Figure 5A,B). 

In addition, we have examined the potency of diphenyl sulfoxide and benzophenone, in which the methyl groups of DMSO and acetone, respectively, were substituted with phenyls. Both molecules increased the adenine aggregation levels (Supplementary Figure S3F,G). Interestingly, while higher hydrogen bond engagement increased the potency of the inhibitor, increased hydrophobicity of the derivate resulted in an opposite effect and accelerated adenine self-assembly. This effect correlates with lower solubility and higher lipophilicity and aromaticity of the molecules (Figure 5A).

### 2.4. Effective Inhibition of Adenine Self-Assembly by USAFDO-38 Correlates with Improved Viability in Yeast

**Figure 6:**
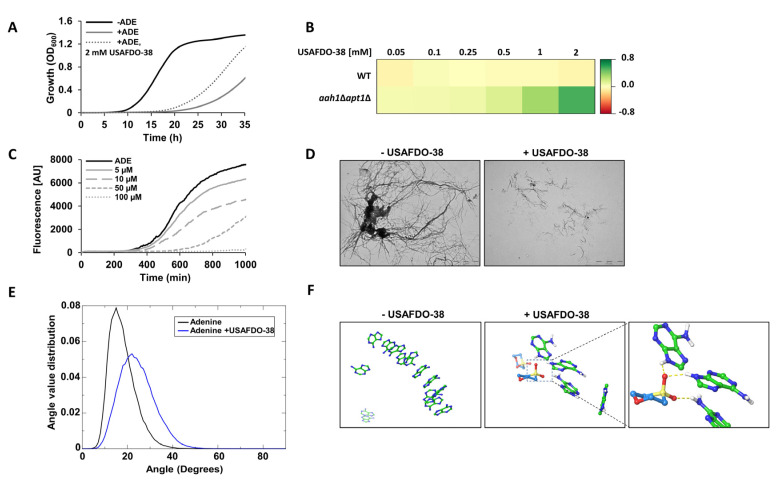
USAFDO-38 inhibits adenine toxicity and self-assembly. (**A**) Growth curves of *aah1*Δ*apt1*Δ cells under the indicated conditions. The absorbance at OD_600_ was measured over time. The results are representative of three independent biological repeats. (**B**) Dose-response heat map representing the difference in optical density between chaperone-supplemented and the control condition (+ADE) when the control cells reached OD_600_ = 0.6. The dose-response heat map was generated based on data shown in the kinetics graphs presented in Supplementary Figure S4A. Green, rescue. Red, toxicity. (**C**) Kinetic analysis of adenine aggregation as measured in a ThT binding assay. Adenine was dissolved at 6 mg/mL in PBS and heated to 90 °C to obtain a monomeric solution, followed by the addition of USAFDO-38 at the indicated concentrations. The control (ADE) was prepared as described in the Materials and Methods. ThT was added to a final concentration of 40 µM, and fluorescence was measured over time with excitation and emission wavelengths of 450 nm and 480 nm, respectively. The results are representative of three independent repeats. (**D**) Representative TEM micrographs of 1 mg/mL adenine in the presence or absence of 10 µM USAFDO-38. Scale bar is 2 µm. The images are representatives of three repeats. (**E**) Distribution of the angles between the normal vectors to the planes of monomers in contact, in the absence and the presence of USAFDO-38, as observed in the MD simulations (Supplementary Figure S4). (**F**) Representative structures obtained from MD simulations in the presence or absence of USAFDO-38. The MD simulation in the presence of USAFDO-38 is magnified and the area is marked with dashed lines. Oxygen atoms are shown in red; sulfur atoms are shown in yellow. Hydrogen bonds are represented as yellow dashed lines.

Out of the DMSO derivatives that were examined, USAFDO-38 emerged as the most potent inhibitor of adenine self-assembly (Figure 5). We thus aimed to investigate its biological activity using the salvage yeast model system. Indeed, we observed a significant dose-dependent rescue of toxicity (Figure 6A,B). Notably, the effective concentration of USAFDO-38 was more than two orders of magnitude lower than that of DMSO (2 mM and 280 mM, respectively) (Figure 2A and Figure 6A,B and Supplementary Figure S4A). In line with the cytotoxicity data, USAFDO-38 effectively inhibited adenine self-assembly rates in vitro in a dose-dependent manner and resulted in shorter and less densely packed fibrils (Figure 6C,D). 

To elucidate the mode of action of USAFDO-38 and provide a qualitative picture of the initial stages of the aggregation process in the absence and presence of the inhibitor, we applied all-atom MD simulations. In our computational setup, a solution of adenine molecules, with and without USAFDO-38, was randomly placed in a simulation box and allowed to evolve over time. Two molecules were defined in contact if the distance between their centers of mass was below 0.6 nm. We then calculated and analyzed the distribution of the angles between the normal vectors to the planes of two adenine molecules in contact. Interestingly, in the presence of USAFDO-38, the angle values were larger and more widely distributed, indicating higher conformational heterogeneity within the ensembles of aggregates compared to pure adenine (Figure 6E,F and Supplementary Figure S4B). Indeed, visual inspection of the aggregate structures observed in snapshots of the simulations indicated that while stacking and elongation of aggregates were possible in the case of pure adenine, USAFDO-38 caused a conformational shift towards smaller and less ordered aggregates where adenine molecules were ‘hijacked’ by the inhibitor. These changes could aptly oppose the initial stages of adenine packing and amyloid-like fibril growth (Figure 6E,F and Supplementary Figure S4B).

In line with the SAR-SPR analysis, the interaction between USAFDO-38 and adenine was mediated by the formation of hydrogen bonds between adenine and the two sulfur-bound oxygen atoms (Figure 6F). Hydrogen bonds involving the intra-ring oxygen atom of USAFDO-38 were also sporadically observed (~6% of the total configurations explored). Despite the significant molecular mass difference compared to USAFDO-38, the hydrophobic compounds DCA and UDCA seem to act in a similar ‘hijacking’ mechanism. Yet, as expected by their hydrophobic nature, their mode of action does not appear to include hydrogen bonding interactions (Supplementary Figure S5).

## 3. Discussion

While amyloid formation has been typically associated with protein misfolding and disease, the extension of the canonical amyloid hypothesis to include metabolites has opened new avenues in the field of molecular self-assembly and amyloid research. Although many properties of metabolite amyloid-like assemblies have been described and demonstrated to resemble those of protein amyloids (Figure 1A), their nature is still not fully comprehended. In this work, we describe the inhibition by chemical chaperones as a common hallmark of protein and metabolite amyloid assemblies. Using a combined in vivo-in vitro-in silico approach, we have investigated the inhibition of adenine self-assembly by chemical chaperones, shedding light on the molecular mechanism underlying this interaction. The chemical chaperones that were examined effectively rescued the toxic phenotypes of adenine accumulation and self-assembly in a cellular model and inhibited metabolite self-assembly in vitro while altering the morphology of the nano-assemblies. Moreover, consistent with the experimental results, analysis of MD simulations indicates that the inhibitors interfere with ordered aggregation mechanisms by hijacking accumulated adenine molecules. The observed experimental and computational analysis indicate a possible similarity with the mechanism of action of the polyphenols epigallocatechin gallate (EGCG) and curcumin and its derivatives, for both protein and metabolite aggregates, that redirect aggregation towards nontoxic, unstructured species [58,59,60,61,62]. We have conducted a SAR-SPR analysis of various DMSO derivatives, pointing to the role of hydrogen bonding in the inhibitory mechanism [63]. We have thus identified USAFDO-38 as a potent inhibitor of adenine self-assembly in vitro and its intracellular cytotoxicity. In the in vitro assays, this molecule functions at the micromolar range in a dose-dependent manner via a mechanism involving, inter alia, hydrogen-bonding interactions, and could thus serve as a scaffold for further optimization and development of novel lead-like molecules [52]. 

Many IEMs result from a single amino acid change in a metabolic enzyme, leading to its misfolding, loss of function and consequent metabolite accumulation. Chemical and pharmacological chaperones emerge as promising therapeutics that stabilize and reactivate the mutant enzyme, hence delaying or curing disease phenotypes [64,65]. For instance, the pharmacological chaperone sapropterin (Kuvan) has already been approved as the first non-dietary treatment for patients suffering from phenylketonuria which results from a single amino acid alteration in phenylalanine hydroxylase (PHA) [66]. Furthermore, chemical chaperones have been demonstrated to enhance the activity of mutated PHA, mutated cystathionine beta-synthase known to be associated with homocystinuria, and mutated branched-chain alpha-ketoacid decarboxylase which is associated with maple syrup urine disease [67,68,69]. Moreover, a recent study has reported the inhibition of hyperoxaluria phenotypes as well as the reduction of endoplasmic reticulum stress and overall cellular oxidative stress by the chemical chaperone 4-phenylbutyric acid [70]. 

Studies examining the potential of chemical chaperones as IEM therapies have so far been exclusively focused on the stabilization of the misfolded enzyme. However, since the yeast model used in this study contains a complete knockout of the open reading frames of *AAH1* and *APT1*, rather than a point mutation, the biological activity of the chemical chaperones in this model cannot be attributed to enzyme stabilization. Furthermore, we report that glycerol, a chemical chaperone previously demonstrated to stabilize PHA [71], inhibits the formation of phenylalanine amyloid assemblies in vitro (Supplementary Figure S6). Therefore, our results suggest a novel model for the mechanism of action of chemical chaperones, that includes the inhibition of toxic metabolite assemblies, alongside facilitating proper enzyme folding. This dual-target approach emerges as a promising strategy for IEM drug design. 

In conclusion, the similarity between the properties of metabolite amyloids and classical protein amyloids, alongside the simplicity of the metabolite model system, allows careful analysis of the functional properties of chemical chaperones. Our results provide a unique insight into the unsolved mode-of-action of these important compounds, which could serve as scaffolds for the design of small molecules that target metabolite self-assembly in pathological conditions such as IEMs.

## 4. Materials and Methods

### 4.1. Yeast Strains and Culture 

The strains used in this work were BY4741 (WT) and the recently published adenine salvage mutant, *aah1*Δ*apt1*Δ, carrying a deletion of both *AAH1* and *APT1*, the orthologs of the human adenosine deaminase and adenine phosphoribosyl transferase, respectively [27]. Strains were cultured in SD medium consisting of a defined mixture of amino acids and nucleobases, with adenine (adenine hemisulfate) either dropped out (−ADE) or supplemented at 2 mg/L (+ADE). 

### 4.2. Materials

Adenine hemisulfate salt ≥99%, TMAO, DMSO, DCA, UDCA, sulfolane ≥99%, benzophenone, diphenyl sulfoxide, and ThT were purchased from Sigma-Aldrich (Rehovot, Israel). 1,4-Thioxane-1,1-dioxide ≥98% and tetrahydrothiophene 1-oxide were purchased from Tzamal D-Chem (Petah Tikva, Israel). ProteoStat dye was purchased from Enzo Life Sciences, Inc. (Farmingdale, NY, USA). DCA, UDCA, 1,4-Thioxane-1,1-dioxide, benzophenone and diphenyl sulfoxide stocks were prepared and diluted in pure DMSO. TMAO and tetrahydrothiophene 1-oxide stocks were prepared and diluted in PBS.

### 4.3. Yeast Growth Assays

Strains were grown overnight at 30 °C in 5 mL SD −ADE medium. For spotting assay, cells were diluted to OD_600_ = 0.6 and were then five-fold serially diluted and spotted on SD media containing 2 mg/L adenine and the indicated concentrations of the chemical chaperones. Plates were incubated at 30 °C for 2 days. For kinetic growth assays, cells were diluted to OD_600_ = 0.01 in a final volume of 200 µL in a 96-well microplate (Greiner Bio-One, Kremsmünster, Austria). The plate was incubated at 30 °C with continuous shaking and OD_600_ was measured using a Tecan SPARK 10M plate reader (Tecan Trading AG, Männedorf, Switzerland). Assays were performed in triplicates and the results displayed are representative of three independent biological repeats. 

### 4.4. Flow Cytometry 

Cells were grown to mid-log phase (OD_600_ = 0.6) under the indicated conditions. One milliliter of the cultured cells was washed with PBS and sonicated for 2 min at 43 kHz (Ultrasonic Cleaner 0.6L 220V DG-1, MRC Labs, Holon, Israel). For each sample, 2 × 10^6^ cells were resuspended in ProteoStat (prepared according to the manufacturer’s instructions) diluted 1:3000 in PBS. Cells were incubated in the dark for 15 min at room temperature. Flow cytometry was performed using Stratedigm S1000Exi flow cytometer and the Cell-CapTure software (Stratedigm, San Jose, CA, USA). Live cells were gated (P1) by forward scatter and side scatter. Fluorescence channels for FITC (530/30) and PE-Cy5 (676/29) were used with a 488 nm laser source. A total of 50,000 events were acquired for each sample. Analyses were performed using the FlowJo software (TreeStar, version 10). The results displayed are representative of three independent biological repeats.

### 4.5. Confocal Microscopy 

Cells were grown to mid-log phase (OD_600_ = 0.6) in SD medium supplemented with 2 mg/L adenine and the indicated concentrations of the chemical chaperones. One milliliter of cells was washed with PBS, sonicated for 2 min at 43 kHz (Ultrasonic Cleaner 0.6L 220V DG-1, MRC Labs, Israel) and resuspended in 50 µL of ProteoStat (prepared according to the manufacturer’s instructions) diluted 1:250 in PBS. Cells were incubated in the dark for 15 min at room temperature. 10 µL of each sample was deposited on polylysine-coated glass slides (Sigma-Aldrich). Cells were imaged using Leica TCS SP8 laser confocal microscope with ×100 1.4 NA oil objectives (Leica Microsystems, Wetzlar, Germany). Excitation and emission wavelengths of 488 nm and 500–600 nm, respectively. The results displayed are representative of three independent biological repeats.

### 4.6. ThT Fluorescence In Vitro Kinetic Assay 

Adenine (6 mg/mL) was dissolved in PBS by heating to 90 °C for 3 h to obtain a monomeric solution. The adenine solution was then mixed with the chemical chaperones at the indicated concentrations in a black clear flat-bottom 96-well microplate (Greiner Bio-One, Kremsmünster, Austria). ThT was added to a final concentration of 40 µM. The control was prepared according to the solvent used for stock preparation: adenine dissolved in PBS was used as a control for the osmolytes, thiophane oxide and sulfolane; adenine in PBS containing 0.25% DMSO for hydrophobic compounds; and adenine in PBS containing 0.1% DMSO for USAFDO-38, benzophenone, and diphenyl sulfoxide. Fluorescence was measured over time using a Tecan SPARK 10 M plate reader (Tecan Trading AG, Männedorf, Switzerland) with excitation and emission wavelengths of 450 nm and 480 nm, respectively. Assays were performed in triplicates, and the results displayed are representative of three independent repeats. 

The relative concentration was calculated after 1000 min of incubation, and the concentration of the inhibitor was divided by the percentage of inhibition after the stated incubation time. The relative concentration represents the minimal concentration required to inhibit 1% of adenine fibrilization (relative con. [mM/1% inhibition]).

### 4.7. ProteoStat Fluorescence In Vitro Kinetic Assay

Adenine (6 mg/mL) was dissolved in PBS by heating to 90 °C for 3 h to obtain a monomeric solution. The adenine solution was then mixed with DMSO or DCA at the indicated concentrations in a black clear flat-bottom 96-well microplate (Greiner Bio-One, Kremsmünster, Austria). The samples were mixed with ProteoStat (prepared according to the manufacturer’s instructions) at a 1:100 ratio. Adenine diluted in PBS was used as a control for DMSO-treated samples, while 0.25% DMSO was supplemented as a control for DCA assays. Fluorescence was measured over time using a Tecan SPARK 10M plate reader (Tecan Trading AG, Männedorf, Switzerland) with excitation and emission wavelengths of 485 nm and 620 nm, respectively. Assays were performed in triplicates, and the results displayed are representative of three independent repeats. 

### 4.8. Transmission Electron Microscopy 

Adenine (1 mg/mL) was dissolved in PBS by heating to 90 °C for 3 h to obtain a monomeric solution. The adenine solution was then mixed with the chemical chaperones at the indicated concentrations. Adenine diluted in PBS served as a control for the osmolyte-treated samples, while 0.005% DMSO was supplemented as a control for samples treated with hydrophobic compounds. The solutions were gradually cooled down and incubated overnight at room temperature to facilitate self-assembly. Subsequently, the samples were vortexed and 10 µL of each was placed on 400-mesh copper grids (Electron Microscopy Sciences, Hatfield, PA, USA). The grids were incubated at room temperature for two minutes, and excess fluids were absorbed. Samples were visualized using a JEOL 1200EX electron microscope operating at 80 kV.

### 4.9. MD Simulations 

MD simulations were performed for the following systems: a solution of pure adenine; a solution containing adenine and USAFDO-38; a solution containing adenine and DCA; and a solution containing adenine and UDCA. The results of the latter two simulations are reported in the Supplementary Materials (Supplementary Figures S4 and S5). The simulation systems were prepared by filling a 100Å^2^ cubic box with 44 molecules of adenine alone, 40 molecules of adenine and 5 molecules of USAFDO-38, 40 molecules of adenine and three molecules of DCA, or 40 molecules of adenine and three molecules of UDCA, respectively. Each box was filled with water and NaCl to reach 150 mM concentration while also ensuring global electroneutrality for each system. The concentration of adenine in the simulated systems corresponds to the experimentally used one (6 mg/mL). Amber force field parameters for adenine, USAFDO-38, DCA, and UDCA were developed using the antechamber program. The procedures were conducted using the General Amber Force Field (GAFF). Atomic partial charges were generated by the Restrained Electrostatic Potential (RESP) approach on Molecular Electrostatic Potential (MEP) calculated by Gaussian09 [72]. In this framework, adenine and the various chemical chaperones were minimized with Gaussian 03 at the RHF/6-31G level. The MEP for each molecule was calculated at the RHF/6-31G* level. 

All MD simulations and standard structural analyses were performed with the AMBER 16 suite of programs, using the TIP3P water model and the CUDA implementation for Graphical Processing Units (GPUs) [73]. Each simulation started with an unrestrained minimization consisting of 2500 steps of steepest descent followed by 2500 steps of conjugate gradient minimization. The minimized systems were then equilibrated at 300 K for 10 ns using Langevin coupling with gamma equal to 1 ps^−1^. The relaxed systems were simulated in the NPT ensemble at 1 atm using Berendsen coupling algorithms [74]. The full particle-mesh Ewald method was used for electrostatics [75]. The SHAKE algorithm was used to constrain all covalent bonds involving hydrogen atoms [76]. A 2 fs time step and a 10 Å cutoff were used for the truncation of van der Waals nonbonded interactions. 

For each system, four independent simulations were performed, starting with different sets of initial velocities and random positioning of the molecules in the box. Only the last 200 ns of each simulation were retained for analysis. For each system, the respective 200 ns trajectories were combined to yield a combined metatrajectory of 800 ns.

## Data Availability

Not applicable.

## Supplementary Materials

The following are available online at https://www.mdpi.com/article/10.3390/ijms22179172/s1.
