# Supplementary material for: Chemical Chaperones Modulate the Formation of Metabolite Assemblies
Source: Int J Mol Sci. 2021 Aug 25;22(17):9172. doi: 10.3390/ijms22179172 (PMC8431448; doi:10.3390/ijms22179172)
Supplement: Supplementary file 1 [file ijms-22-09172-s001.zip › ijms-1353965-supplementary.pdf]

Supplementary Materials

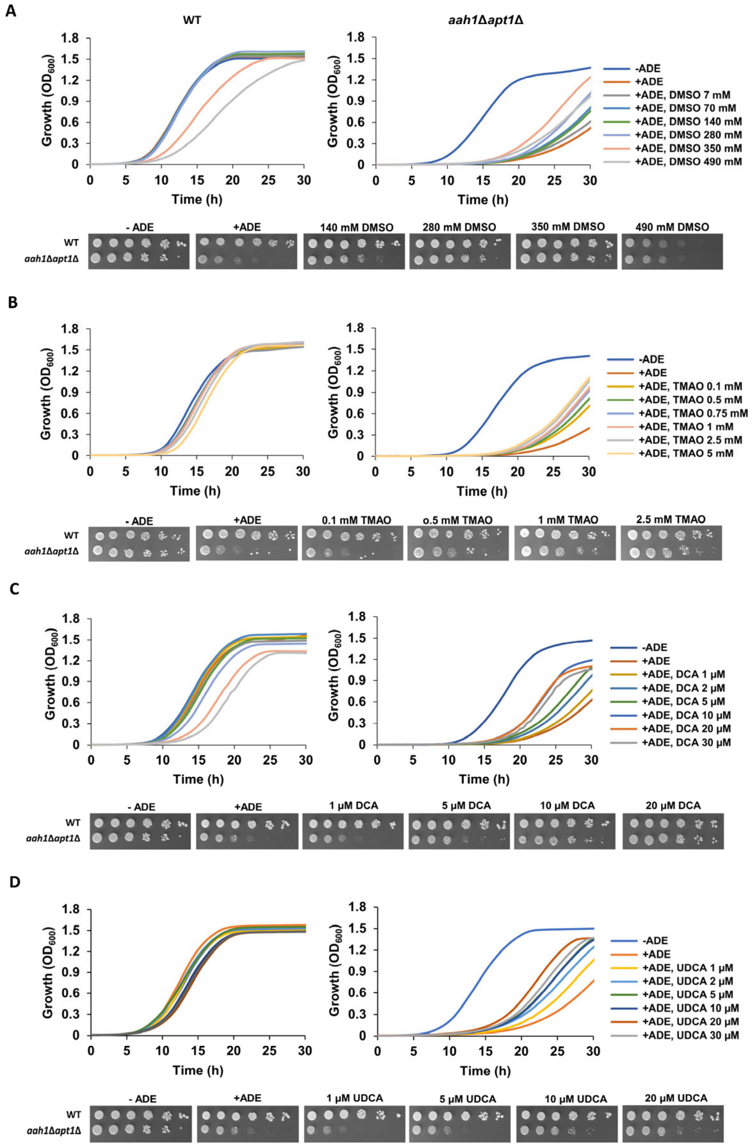

Supplementary Figure S1. Inhibition of adenine toxicity by different chemical chaperones in yeast. **Upper panel**: Growth curves of wild type (WT) and *aah1*Δ*apt1*Δ cells under the indicated conditions. The absorbance at OD_{600} was measured over time. The results are representative of three independent biological repeats. **Lower panel**: WT and *aah1*Δ*apt1*Δ strains were serially diluted and spotted on synthetic defined (SD) media lacking adenine (-ADE), SD media with adenine supplemented at 2 mg/L as a control for DMSO and TMAO, or with 2 mg/L adenine and 0.1% DMSO as a control for DCA and UDCA (+ADE), and SD containing 2 mg/L adenine and the indicated concentrations of the chemical chaperons. Cells were grown at 30°C for 2 days. (**A**) DMSO; (**B**) TMAO; (**C**) DCA; (**D**) UDCA.

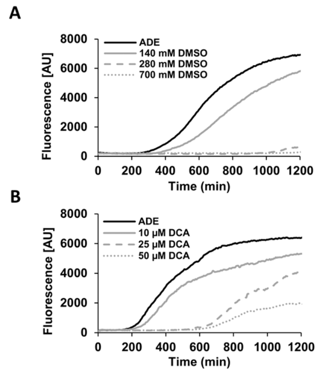

Supplementary Figure S2. Inhibition of adenine fibril formation by different chemical chaperones. ProteoStat fluorescence assay was performed with 6 mg/ml adenine. To obtain a monomeric solution, adenine was dissolved in PBS at 90°C, followed by the addition of the indicated concentrations of each chemical chaperone. The control (ADE) was prepared as described in the Materials and Methods. ProteoStat was added as described in the Materials and Methods. ProteoStat emission data at 620 nm (excitation at 485 nm) was measured over time using the Tecan SPARK 10M plate reader. The results are representative of three experiments. (**A**) DMSO; (**B**) DCA.

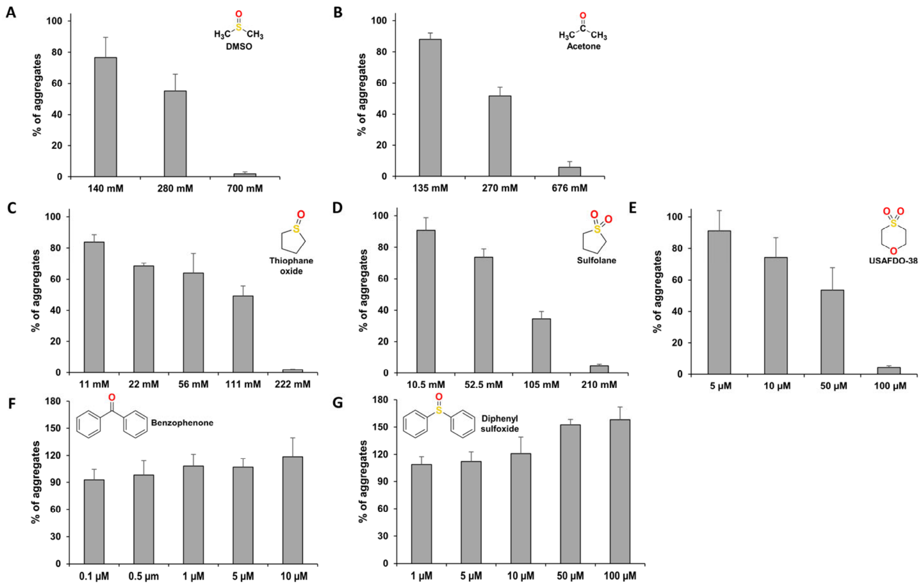

Supplementary Figure S3. Inhibition of adenine fibrils formation by different DMSO derivatives. Kinetic analysis of adenine aggregation as measured in a ThT binding assay. Adenine was dissolved at 6 mg/ml in PBS and heated to 90°C to obtain a monomeric solution, followed by the addition of DMSO or its derivatives at the indicated concentrations. The control was prepared as described in the Materials and Methods. ThT was added to a final concentration of 40 µM, and fluorescence was measured over time with excitation and emission wavelengths of 450 nm and 480 nm, respectively. Endpoint fluorescence readings (after 1000 minutes) are presented as the percentage of aggregation in the presence of the indicated concentration of the derivatives compared to the control. The results are representative of three independent repeats. (**A**) DMSO; (**B**) Acetone; (**C**) Thiophane oxide; (**D**) Sulfolane; (**E**) USAFDO-38; (**F**) Benzophenone; (**G**) Diphenyl sulfoxide. Oxygen atoms are shown in red; sulfur atoms are shown in yellow.

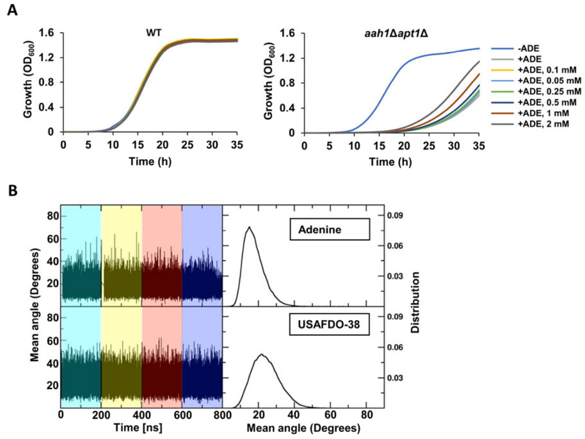

Supplementary Figure S4. USAFDO-38 inhibits adenine toxicity and fibril formation. (**A**) Growth curves of wild type (WT) and *aah1Δapt1Δ* cells under the indicated conditions. The absorbance at OD_{600} was measured over time. The results are representative of three independent biological repeats**.** (**B**) Left: time evolution of the mean angle between the planes of the adenines calculated along MD simulations. The color shading indicates the different independent trajectories that were combined for analysis. Right: distribution of the mean angle values. The time evolution and the distribution for each system are shown on the same line. From top to bottom: adenine alone; adenine and USAFDO-38.

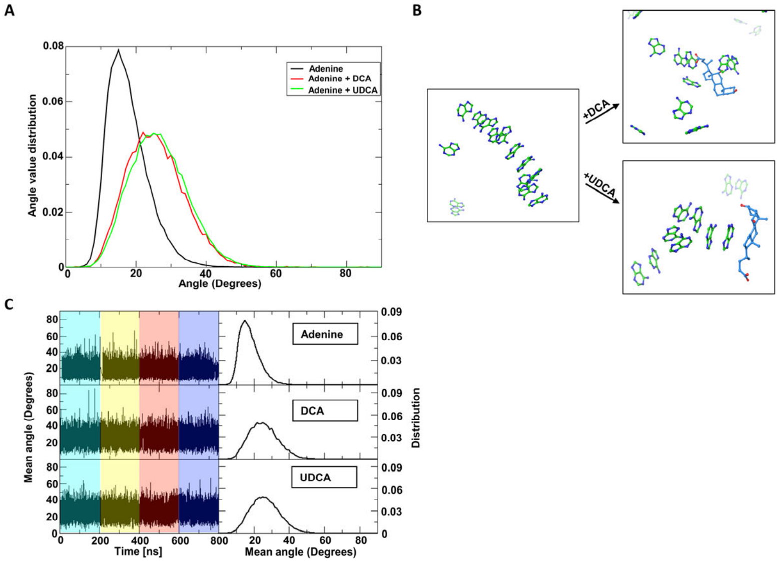

Supplementary Figure S5. Mechanism of action of the bile acids DCA and UDCA. (**A**) Distribution of the angles between the normal vectors to the planes of monomers in contact in the absence and in the presence of the chemical chaperones as observed in the MD simulations. (**B**) Representative structures obtained from MD simulations in the control (left) and upon the addition of the chemical chaperones (right). (**C**) Left: time evolution of the mean angle between the planes of the adenines calculated along MD simulations. The color shading indicates the different independent trajectories that were combined for analysis. Right: distribution of the mean angle values. The time evolution and the distribution for each system are shown on the same line. From top to bottom: adenine alone; adenine and DCA; adenine and UDCA.

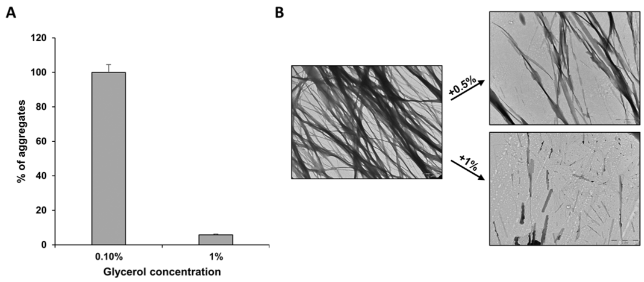

Supplementary Figure S6. Inhibition of phenylalanine fibrils formation by glycerol. (**A**) Endpoint analysis of phenylalanine aggregation as measured in a turbidity assay. Phenylalanine was dissolved at 37 mg/ml in PBS and heated to 90°C to obtain a monomeric solution, followed by the addition of glycerol at the indicated concentrations. PBS was used as the control. Turbidity was measured overtime at a wavelength of 405 nm. Endpoint readings (after 200 minutes) are presented as a percentage of aggregation in the presence of the indicated concentration of glycerol compared to the control. The results are representative of three independent repeats. (**B**) Representative TEM micrographs of 25 mg/ml phenylalanine in the presence or absence of the indicated concentrations of glycerol. Scale bar is 2 μm. The images are representatives of three independent repeats.
